# Cannabinoid Combination Induces Cytoplasmic Vacuolation in MCF-7 Breast Cancer Cells

**DOI:** 10.3390/molecules25204682

**Published:** 2020-10-14

**Authors:** Recardia Schoeman, Natasha Beukes, Carminita Frost

**Affiliations:** Department of Biochemistry and Microbiology, Nelson Mandela University, P.O. Box 77000, Port Elizabeth 6031, South Africa; recardia23@outlook.com (R.S.); natasha.beukes@mandela.ac.za (N.B.)

**Keywords:** breast cancer, cannabinoids, combination studies, anti-cancer, cytoplasmic vacuolation

## Abstract

This study evaluated the synergistic anti-cancer potential of cannabinoid combinations across the MDA-MB-231 and MCF-7 human breast cancer cell lines. Cannabinoids were combined and their synergistic interactions were evaluated using median effect analysis. The most promising cannabinoid combination (C6) consisted of tetrahydrocannabinol, cannabigerol (CBG), cannabinol (CBN), and cannabidiol (CBD), and displayed favorable dose reduction indices and limited cytotoxicity against the non-cancerous breast cell line, MCF-10A. C6 exerted its effects in the MCF-7 cell line by inducing cell cycle arrest in the G_2_ phase, followed by the induction of apoptosis. Morphological observations indicated the induction of cytoplasmic vacuolation, with further investigation suggesting that the vacuole membrane was derived from the endoplasmic reticulum. In addition, lipid accumulation, increased lysosome size, and significant increases in the endoplasmic reticulum chaperone protein glucose-regulated protein 78 (GRP78) expression were also observed. The selectivity and ability of cannabinoids to halt cancer cell proliferation via pathways resembling apoptosis, autophagy, and paraptosis shows promise for cannabinoid use in standardized breast cancer treatment.

## 1. Introduction

*Cannabis sativa* L. has been used for centuries in the treatment of various ailments. One particular group of compounds produced by this plant, the C_21_ terpenophenolics (referred to as cannabinoids), are well known for their vast range of bioactivity [1,2]. There are several types of cannabinoids: endocannabinoids; synthetically synthesized cannabinoids; and phytocannabinoids, which specifically refer to the cannabinoids obtained from cannabis plants [3,4]. The most abundant and psychoactive phytocannabinoid is Δ^9^-tetrahydrocannabinol [1,5]. Other well-known phytocannabinoids include cannabigerol (CBG), cannabinol (CBN), cannabidiol (CBD), cannabichromeme (CBC), and cannabicyclol (CBL), amongst others [2]. Phytocannabinoids mimic endogenous cannabinoids by activating cannabinoid receptors. Depending on the cell type, phytocannabinoids have been documented to modulate cell proliferation, differentiation, and death [6,7]. These properties have gained traction in the cancer field, since the activation of cannabinoid receptor can be exploited to influence several hallmarks of tumor progression. The anti-cancer effects of phytocannabinoids have been observed in several cancer types, including gliomas and carcinomas of the skin, liver, colon, prostate, and breast [8,9,10,11,12]. Breast cancer is particularly difficult to treat due to its heterogeneity [13]. Breast cancer cells are heterogenous and are mainly classified by the expression of the hormone receptors (estrogen and progesterone) and epidermal growth factor receptor 2 (HER2). Breast cancer cells that lack these receptors are referred to as triple-negative breast cancer (TNBC). TNBCs are aggressive and notoriously difficult to treat due to their lack of drug receptor targets. Studies have shown that phytocannabinoids are effective against various breast cancer subtypes as they induce cell cycle arrest and cell death via pathways such as apoptosis and autophagy [14,15,16,17].

Although conventional chemotherapeutic agents exist, patients often experience side-effects that influence their quality of life [18,19]. In addition, cancers often acquire resistance mechanisms to evade cell death pathways, rendering the chemotherapeutic agent obsolete [20,21]. Several studies have evaluated the use of drug combinations to overcome these chemotherapy-associated problems. Some advantages, including a reduction in the required dose [22,23], minimal potential to induce toxicity in the host, a reduction in the cost associated with therapy [23], and the minimal risk of developing drug resistance [24,25] have been associated with the various studies. Consequently, this study aimed to investigate the potential use of cannabinoid combinations to amplify therapeutic efficacy by simultaneously activating multiple anti-cancer mechanisms in breast cancer cell lines. In addition, we identified the mechanism of action of a promising synergistic cannabinoid combination.

## 2. Results

### 2.1. Combination Studies

Prior to evaluating the efficacy of cannabinoid combinations on breast cancer cells, the cytotoxicity of the individual cannabinoids was determined. In addition to the IC_50_ values, the IC_75_ and IC_90_ values were determined to evaluate if the cannabinoids would retain their selectivity for cancerous cells at higher treatment concentrations (Table 1).

The IC values between cannabinoids were significant within a cell line (data not shown), while no significance was observed between the two cell lines. Although not significant, the MD Anderson-Metastatic Breast-231 (MDA-MB-231) triple negative breast cancer (TNBC) cells had a higher susceptibility to the individual cannabinoid treatments when compared to the Michigan Cancer Foundation-7 (MCF-7) breast cancer cells (ER^+^, PR^+^, and HER2^+^), as indicated by the inhibitory concentrations (Table 1). Despite receptor expression being the main difference between the cell lines, the order of cytotoxicity (IC_50_) of the cannabinoids remained the same in both the cell lines: CBD > CBN > CBG > THC. The next step was to determine the effects of various cannabinoid combinations. The IC_50_ values of the individual cannabinoids were used to combine the cannabinoids at equipotent ratios at double their respective IC_50_:IC_50_ concentrations (Table 2), as recommended by Chou [26].

**Figure 1:**
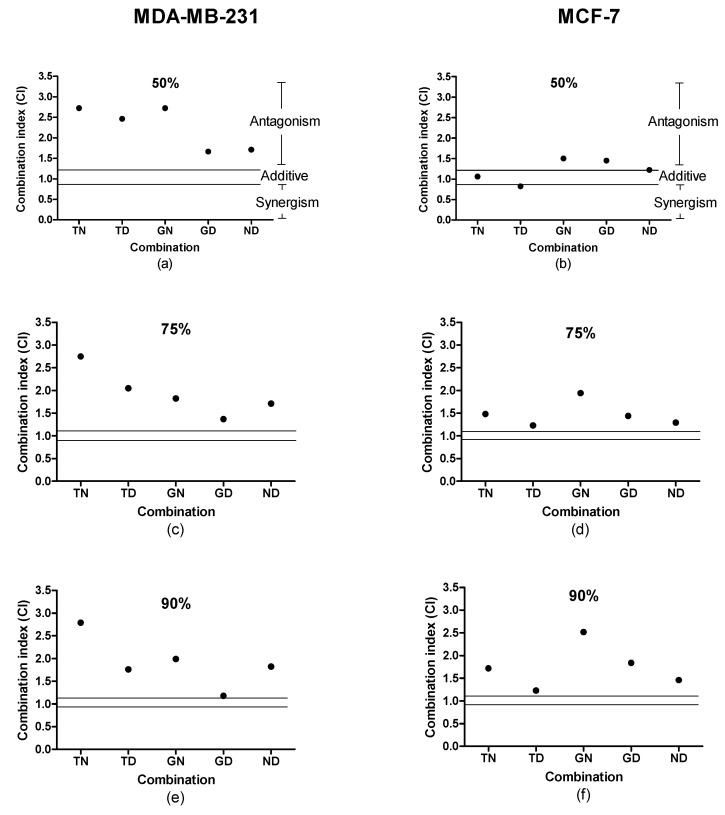
Combination index (CI) values were calculated for the respective two-cannabinoid combinations determined in the MDA-MB-231 (**a**,**c**,**e**) and MCF-7 (**b**,**d**,**f**) cell lines at 50% (**a**,**b**), 75% (**c**,**d**), and 90% (**e**,**f**). CI signifies the combination index at 50%, 75%, and 90% inhibition of the cell population, where CI = [1/Dx)1] + [2/Dx)2], where Dx = Dm[fa/(1-fa)]1/m. A CI < 1 indicates synergism, CI = 1 indicates an additive effect, and CI > 1 indicates antagonism. T, THC; G, CBG; N, CBN; D, CBD.

Despite the inhibitory concentrations of the individual cannabinoids being lower in the MDA-MB-231 cell line, the inhibitory concentrations of the combinations of two cannabinoids were generally lower in the MCF-7 cell line. It should be highlighted that the two combinations with the lowest IC values in the MCF-7 cell line were combinations containing THC, while the opposite was obtained in the MDA-MB-231 cell line. The determination of the combination index values associated with the two-cannabinoid combinations highlighted that the majority of the combinations were antagonistic across the two cell lines (Figure 1), with the exception of the THC:CBD combination, which displayed a synergistic interaction in the MCF-7 cells at a 50% growth inhibition.

**Figure 2:**
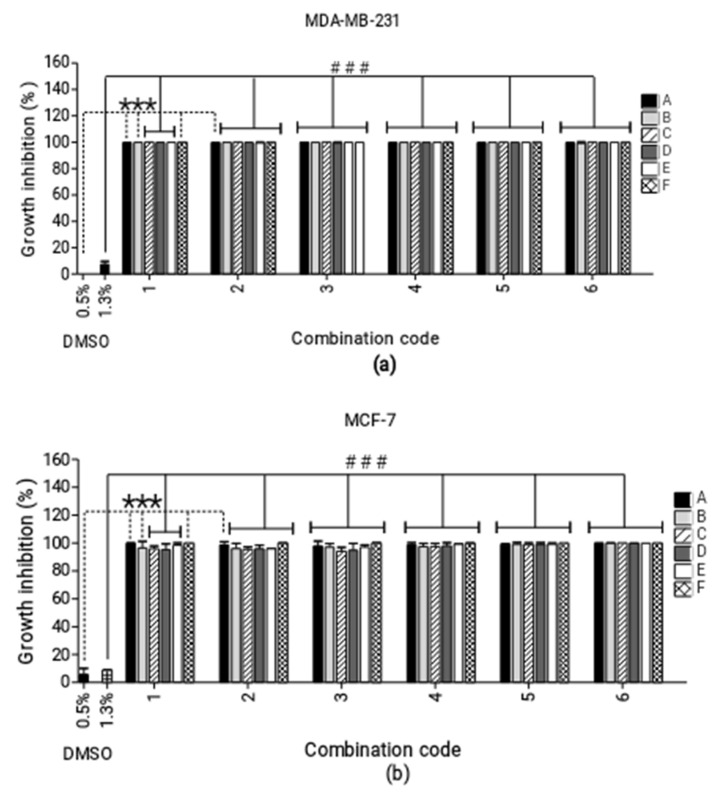
Screening of combinations consisting of four cannabinoids. Percentage growth inhibition induced by various ratios of four-cannabinoid combinations against the (**a**) MDA-MB-231 and (**b**) MCF-7 breast cancer cell lines. *** *p* < 0.0001 relative to 0.5% dimethyl sulfoxide (DMSO) vehicle control and ### *p* < 0.001 relative to 1% DMSO vehicle control. The four-cannabinoid combinations—i.e., A1–F6—are described in Figure 2.

Screening four-cannabinoid combinations (Figure 2) yielded highly cytotoxic growth inhibition percentages ranging between 94% and 100% across both cell lines.

**Figure 3:**
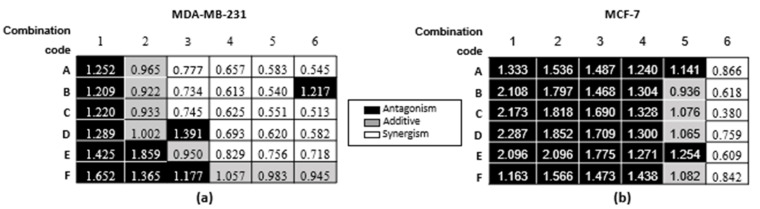
Combination index (CI) values were calculated of each cannabinoid combination at the respective growth inhibitions induced in the (**a**) MDA-MB-231 and (**b**) MCF-7 cell lines.

In order to select the most promising combinations for each cell line, the preliminary combination index values were calculated for each of the combinations (Figure 3).

**Figure 4:**
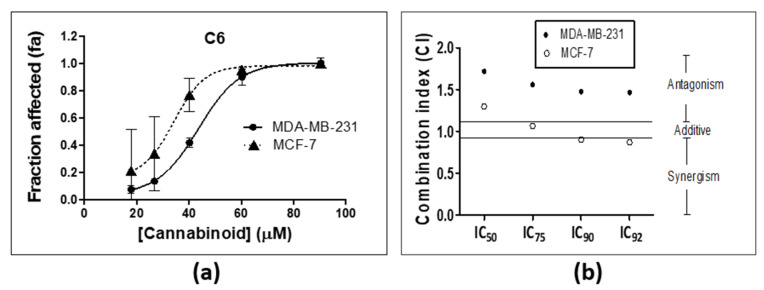
Evaluating the efficacy of C6 across two breast cancer cell lines. (**a**) Dose response curves of C6 in the MDA-MB-231 and MCF-7 cell lines, (**b**) CI value of C6 determined at the selected percentages of growth inhibition in the MDA-MB-231 and MCF-7 cell lines.

As can be seen from Figure 3, several combinations showed promise. The most promising synergistic combination in both cell lines was C6. Dose response curves for C6 were constructed against both cell lines (Figure 4a) to determine the IC values of the combination and subsequently calculate the combination index at the selected growth inhibition percentages (Figure 4b). The CI values for C6 indicated antagonism in the MDA-MB-231 cell line at the selected effect levels, while additivity and slight synergism were observed in the MCF-7 cell line (Figure 4b).

**Figure 5:**
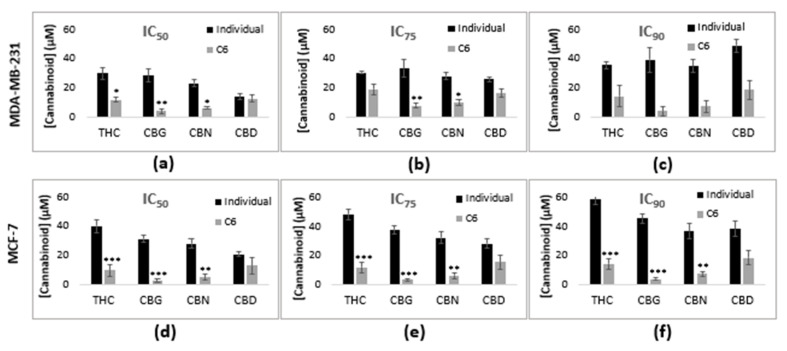
Comparison of the concentration of each cannabinoid, individually and in combination, required to induce a (**a**,**d**) 50%, (**b**,**e**) 75%, and (**c**,**f**) 90% growth inhibition in the (**a**–**c**) MDA-MB-231 and (**d**–**f**) MCF-7 cell lines. * *p* < 0.05, ** *p* < 0.01, *** *p* < 0.001 relative to the respective cannabinoids used individually.

The IC values of the C6 cannabinoid combination were compared against the IC values generated by the individual cannabinoids (Figure 5).

C6 significantly reduced the required doses by 1.4 to 11.6-fold of each respective cannabinoid in the combination, when compared to the concentration of individual cannabinoids required to induce the same percentage growth inhibition. This dose reduction for each cannabinoid in the C6 combination was more significant in the MCF-7 cell line (Figure 5).

### 2.2. Cytotoxicity in Non-Cancerous Cells

**Figure 6:**
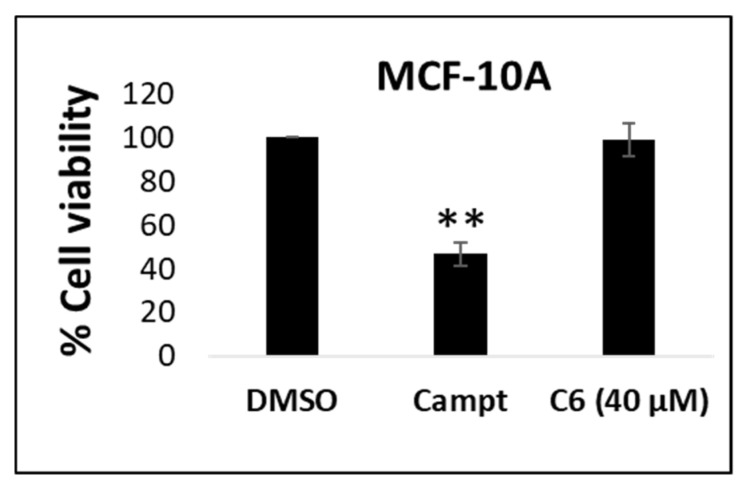
Screening of C6 in the non-cancerous breast cell line, MCF-10A, to evaluate its selectivity for breast cancer cells. ** *p* < 0.01. Camptothecin (5.74 µM) was used as a positive control to induce growth inhibition. Campt, camptothecin.

The selectivity of C6 for breast cancer cells was evaluated by screening the IC_90_ of the combination against the non-cancerous breast cell line, MCF-10A (Figure 6). The known anti-cancer agent, camptothecin, was used as a positive control during the evaluation of cytotoxicity in the MCF-10A cell line. C6 displayed some of the advantages of drug combinations, showing no significant reduction in cell viability (1%) in the non-cancerous MCF-10A breast cell line (Figure 6).

Mechanism studies were to be completed with the C6 cannabinoid combination in the cell line, which displayed a synergistic interaction; however, the synergism observed was not promising, since the C6 combination only yielded a synergistic interaction at a very specific effect level, rather than at multiple effect levels. Alternatively, mechanism studies were completed for C6, based on the induction of morphological changes in the MCF-7 cell line (Section 2.4).

### 2.3. Cell Cycle Arrest and Apoptosis

**Figure 7:**
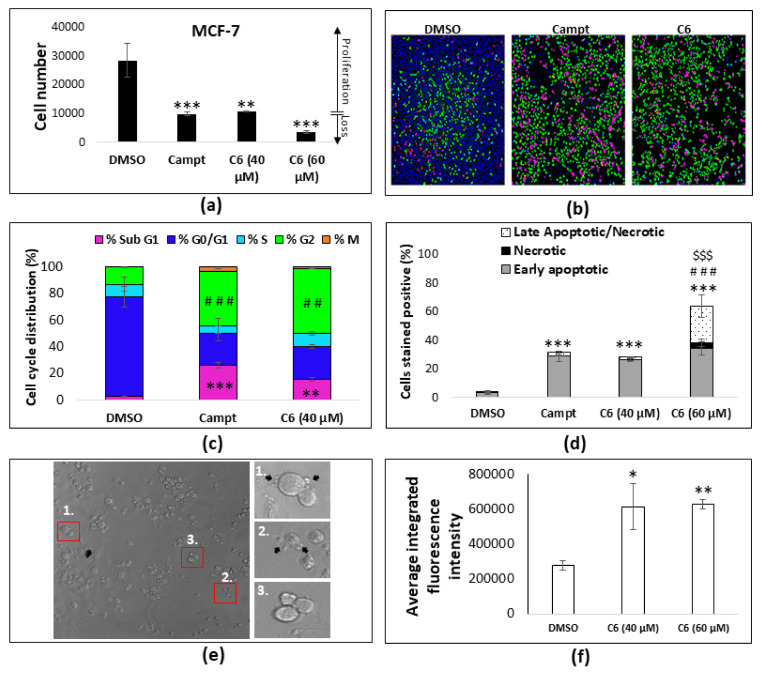
Mechanism of action through which C6 induces its anti-proliferative action. (**a**) Cell number determination in the MCF-7 cell after treatment at two concentrations, using the Hoechst 33,342 staining and image acquisition method. ** *p* < 0.01, *** *p* < 0.001 relative to DMSO vehicle control. (**b**) Representative images acquired during cell cycle analysis, where each color represents the phase of the cell cycle assigned to the specific cell based on the intensity of the Hoechst 33,342 staining. (**c**) Quantitative analysis of the percentage of the cell population in each phase of the cell cycle. Camptothecin (5.74 µM) was used as a positive control for cell cycle arrest in the G2 phase. ** *p* < 0.01, *** *p* < 0.001 relative to the DMSO vehicle control of % Sub G1; ## *p* < 0.01, ### *p* < 0.001 relative to DMSO vehicle control of %G2 using a one-way ANOVA with post hoc Tukey test (n = 3). (**d**) Quantitative analysis of the cell population undergoing apoptosis or necrosis. *** *p* < 0.001 relative to the % of early apoptotic cells of DMSO vehicle control; ### *p* < 0.001 relative to the % of necrotic cells of DMSO vehicle control and $$$ *p* < 0.001 relative to the % of late apoptotic cells of the DMSO vehicle control using a one-way ANOVA with a post hoc Tukey test (*n* = 3). (**e**) Morphological changes induced by C6 showing membrane blebbing (black arrows) and cell shrinkage. (**f**) Quantification of the average integrated nuclear fluorescence intensity. * *p* < 0.05, ** *p* < 0.01, relative to the DMSO vehicle control. Campt, camptothecin.

When evaluating the MCF-7 cell number at 40 and 60 µM (the total concentration of all the cannabinoids comprising the C6 combination) (Figure 7a), the cell number after 40 µM of treatment did not deviate from the 10,000 cells that were initially seeded, indicating that the C6 induced a cytostatic effect. At a concentration of 60 µM of C6, the cell number decreased below the initial 10,000 cells seeded, indicating the induction of a cytotoxic effect at higher concentrations. The cytostatic and cytotoxic effects were further analyzed through cell cycle analysis, and the induction of apoptosis through quantifying the translocation of phosphatidylserine. Cannabinoids are known to induce cell cycle arrest at both the G_0_/G_1_ and G_2_/M checkpoints [27,28,29]. Previous studies have utilized flow cytometry to analyze cell cycle arrest; however, this technique cannot distinguish between cell cycle arrest in the G_2_ and M phase, while in this study an image-based assay was employed, allowing the distinction between the G_2_ and M phases (Figure 7b,c).

The cell cycle phase distribution in the MCF-7 cells showed a significant increase (*p* < 0.01) in the G_2_ phase, with a shift from 13% in the DMSO vehicle control to 48% in cells treated with 40 µM of C6 (Figure 7c). In addition, there were significant increases (*p* < 0.01) in the percentage of cells in the Sub-G1 phase, indicative of apoptotic cells. Cells were stained with Annexin V, which specifically binds to phosphatidylserine that translocate from the inner to the outer leaflet of the cell membrane when apoptosis is initiated. Cells were simultaneously stained with propidium iodide, an impermeable DNA dye that only stains positive if the cell membrane integrity is disrupted. Although treating the MCF-7 cells with 40 µM of C6 did not result in a loss of cell number, indicating cell death (Figure 7a), the evaluation of phosphatidylserine translocation indicated that at least 28% of the cells were in the early phase of apoptosis (Figure 7d). At the higher concentration of 60 µM, the cytotoxic effects of C6 were enhanced, producing a significant increase in the late apoptotic phase. A total of 60% of the cell population underwent apoptosis in both the early and late phases, whereas the percentage of cells that stained positive for propidium iodide only (necrotic) were limited. After treatment, the MCF-7 cells also underwent morphological changes associated with the induction of apoptosis, such as membrane blebbing, cell shrinkage (Figure 7e), and the condensation of the nucleus. When staining the nucleus with a fluorescent stain, the average integrated fluorescence intensity increases when the nuclear material is condensed [30]. This was observed after the Hoechst 33,342 staining of the MCF-7 nuclei (Figure 7f), further supporting the induction of apoptosis.

### 2.4. Lipid Droplet Accumulation

**Figure 8:**
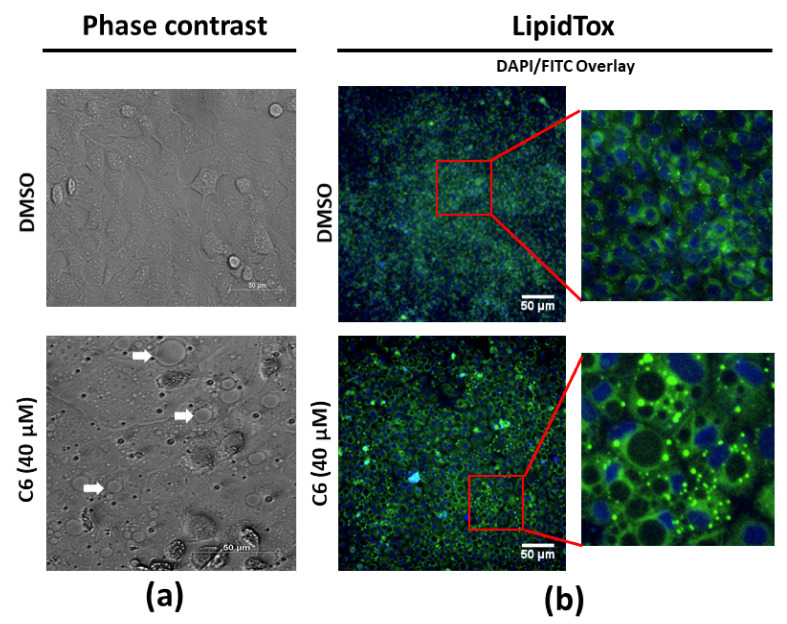
Evaluating lipid droplet accumulation in the MCF-7 cell line. (**a**) Morphological changes induced by C6 observed with phase contrast micrographs; white arrows indicate structures of interest. (**b**) Typical micrographs obtained for MCF-7 cells stained with HCS LipidTOX^TM^ Green neutral lipid stain after treatment with C6.

The morphological changes observed in the MCF-7 cells treated with C6 showed the presence of spherical structures resembling lipid droplets (Figure 8a). A previous study reported that, after epithelial-mesenchymal transition, breast cancer cells can be terminally differentiated into adipocytes when co-treated with a peroxisome proliferator-activated receptor gamma (PPARγ) agonist (rosiglitazone) and mitogen-activated protein kinase kinase (MEK) inhibitor (trametinib) [31]. Studies specifically investigating the phytocannabinoids used in this study have reported the activation of PPARγ by THC [32], CBG [33], and CBD [32]. In addition, CBN [34,35] and THC [36] inhibit the MEK pathway. Therefore, a combination like C6, comprising THC, CBG, CBN, and CBD, theoretically had the potential to induce the differentiation of breast cancer cells into adipocytes. MCF-7 cells were therefore stained with a neutral lipid stain (Figure 8b). The results indicated that there was an accumulation of neutral lipids in the treated cells; however, the initial structures believed to be lipid droplets remained unstained. It was therefore hypothesized that these spherical structures could be cytoplasmic vacuoles, induced by processes such as autophagy and paraptosis.

### 2.5. Autophagy and Paraptosis

**Figure 9:**
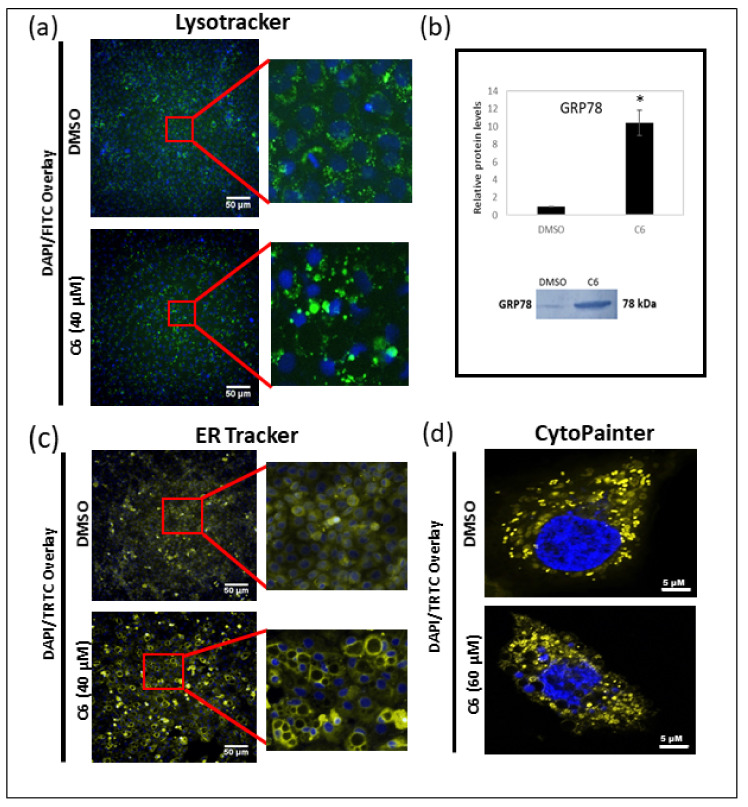
**Evaluation of the MCF-7 organelle structures and GRP78 protein expression.** (**a**) Representative micrographs of MCF-7 cells stained with Lysotracker^TM^ for lysosomes, (**b**) Western blot analysis representing the relative protein expression levels of GRP78 in MCF-7 cells treated with C6 (40 µM) and representative membrane showing band intensities, * *p* < 0.05 relative to the DMSO vehicle control. Representative micrographs of MCF-7 vehicle control and C6-treated cells stained with (**c**) ER Tracker^TM^ to visualize the endoplasmic reticulum membrane and (**d**) CytoPainter^TM^ to visualize the mitochondria.

Consequently, the induction of autophagy was indirectly evaluated through staining with LysoTracker^TM^ Green to visualize acidic organelles—e.g., late autophagic vesicles (Figure 9a). The DMSO vehicle control displayed several lysosomes with a similar size that were distributed throughout the cell. The MCF-7 cells treated with C6 displayed fewer lysosomes; however, they were larger in size and located close to the nucleus of the cell.

A study by Shrivastava et al. [17] showed that CBD coordinates a cross-talk between apoptosis and autophagy. The study indicated that one link between the two pathways was the induction of endoplasmic reticulum stress, therefore, the GRP78 protein expression levels were quantified (Figure 9b). There was a significant increase in the relative GRP78 protein levels in MCF-7 cells treated with 40 µM of C6, indicating that endoplasmic reticulum stress may be a possible link for the induction of both apoptosis and autophagy.

Since cytoplasmic vacuolation can be induced by both autophagy and paraptosis, the markers of paraptosis were also assessed. Endoplasmic reticulum staining indicated an increase in fluorescence surrounding the vacuole membrane, indicative of the vacuole membrane being derived from the endoplasmic reticulum membrane (Figure 9c). In addition, the mitochondrial staining showed changes in the mitochondrial structure from a fibrous to a rounded morphology, associated with mitochondrial dilation. Nuclear fragmentation, a common marker of apoptosis, was also evident in the confocal images of MCF-7 cells treated with 60 µM of C6 (Figure 9d). Therefore, markers of both cell death pathways, apoptosis and paraptosis, were evident.

## 3. Discussion

The heterogeneity within breast cancers has raised several calls for patient-specific treatments to improve prognosis [37]. The MDA-MB-231 and MCF-7 cell lines differ in their phenotypic and genotypic levels. The major differences include the expression of the hormone (estrogen and progesterone) receptors and the HER2 receptor by the MCF-7 cells, while the MDA-MB-231 cell line is representative of triple-negative breast cancer [38]. THC has been reported to suppress the ERα-mediated proliferation of MCF-7 cells via the upregulation of the ER_β_ repressor [39,40]. This could explain why the two cannabinoid combinations containing THC had the lowest IC values amongst the combinations tested (Table 2). In addition, the binding of cannabinoids to the CB_2_ receptor disrupts the HER2/CB_2_ heterodimer formation, inhibiting proliferation via the PI3K/Akt pathway [41]. Since the C6 combination comprises four cannabinoids that can all bind to the CB2 receptor [42], their anti-proliferative effects in the MCF-7 cells would be amplified when cannabinoids are combined. The use of cannabinoid combinations to simultaneously target the multiple receptors expressed by the MCF-7 cell line that are lacking in the MDA-MB-231 cell line could possibly explain the lower inhibitory concentrations observed for the cannabinoid combinations (Table 2) and why the dose reduction was more significant in the MCF-7 cell line (Figure 5). Therefore, this study provided evidence for the potential of cannabinoid combinations to be tailored to the molecular targets uniquely expressed within the patient’s tumor, improving the treatment efficacy and prognosis.

It could be hypothesized that the spherical structures observed (Figure 8a) were vesicles derived from the cell membrane in the form of endosome-associated clathrin-coated buds (approximately 60 nm in diameter) or plasma membrane-derived clathrin-coated vesicles (approximately 100 nm in diameter) [43]. Clathrin-coated pits generally occupy 1% to 2% of the cell surface area, range between 60 and 100 nm in diameter, and have an estimated lifetime of 1 to 2 min [43,44]. Clathrin-independent plasma membrane invaginations also exist and are referred to as caveolae [45]. Caveolae are enriched in lipid rafts of cholesterol and sphingolipids such as ceramide, which are neutral lipids [46]. The spherical structures observed in this study occupied more than 2% of the cell surface and were larger than 100 nm (Figure 8a). During the imaging of the MCF-7 cells, no membrane dynamics were visible, and the spherical structures remained the same size, indicating that the structures observed had a lifetime exceeding 2 min. Since caveolae contain neutral lipids, an increased fluorescence intensity should be evident within the membrane of the spherical structure when staining with LipidTox^TM^ if these structures were derived from the cell membrane in the form of caveolae; however, this was not observed (Figure 8b). In addition, an increased fluorescence intensity was observed when staining with ER-tracker^TM^ (Figure 9c), indicating that the membrane of the spherical structure was derived from the endoplasmic reticulum rather than from the cell membrane. This suggests that it is unlikely that the spherical structures observed in this investigation could be derived from the cell membrane. However, to certainly exclude this possibility future studies could evaluate the localization of clathrin (for clathrin-dependent structures) and caveolin (for clathrin-independent caveolae) in relation to the spherical structures. The anti-proliferative effects of cannabinoids are well-documented [12,14,47,48]. Specifically, in breast cancer cells, the phase of cell cycle arrest is dependent on the type of cannabinoid used in treatment. When bound to CB_2_, THC downregulates cell division control 2 (Cdc2), inducing cell cycle arrest in the G_2_/M [28]. CBD has been shown to induce cell cycle arrest in the G_1_/S phase [49], mediated via the CB_1_ receptor [12], while cannabinoid combinations have been found to induce simultaneous arrest in all phases of the cell cycle [50]. In the MCF-7 cell line, the CB_2_ receptor is more prominently expressed than CB_1_ [28]; therefore, it could be eluded in this study that the G_2_ arrest induced by C6 (Figure 7c) was mediated via the CB_2_ receptor.

Cannabinoids commonly induce cell death via two pathways: autophagy and apoptosis. Autophagy is a conserved cellular process in which the cytoplasmic contents are sequestered into double-membraned vesicles (autophagosomes) and fused with lysosomes for degradation or recycling. Therefore, an increase in the lysosomal content often serves as an indirect method of measuring the induction of autophagy (Figure 9a). The cannabinoid-induced activation of autophagy is often mediated by the induction of endoplasmic reticulum stress [14,51,52]. The inhibition of the mammalian target of rapamycin complex 1 (mTORC1) is a key step in the activation of autophagy [53]. THC induces endoplasmic reticulum stress by triggering the accumulation of ceramide and the phosphorylation of the eukaryotic translation initiation factor 2α. This activates the endoplasmic reticulum stress stress response, which leads to the tribble homolog 3-dependent inhibition of the protein kinase B/mTORC1 pathway [52], resulting in autophagy [51]. Since GRP78 is a marker of endoplasmic reticulum stress and was significantly increased after the treatment with C6 (Figure 9b), this could be an indication that C6 induced endoplasmic reticulum stress-mediated autophagy (Figure 9a,b). Furthermore, the initiation of autophagy is commonly associated with lipid droplet inclusion into autophagosomes and subsequent degradation by lysosomes (lipophagy) [54,55]; however, in this study an accumulation of lipid droplets was observed. The small guanosine triphosphatase Rab7 is indispensable for lipophagy [56] and is associated with lipid droplets and lysosomes under starvation conditions [57]. When Rab7 is silenced, there is an accumulation of lipid droplets [56]. The colocalization of Rab7 and CB_2_ on the lysosomal membrane has been reported [58]; however, it is yet to be established whether the colocalization plays a role in the attenuation of lipophagy and the subsequent accumulation of lipid droplets.

Studies have documented the accumulation of lipids in MCF-7 cells in response to various stimuli, which include the presence of peroxisome proliferator-activated receptor gamma (PPARγ) agonists [32]. Cannabinoids, specifically THC and CBD, have been reported to activate PPARγ, possibly explaining the increase in lipid accumulation observed in the MCF-7 cells. Moreover, PPARγ activations has been linked to pro-apoptotic signaling in MCF-7 cells [59]. This supports the lipid accumulation (Figure 8b), significant increases in phosphatidylserine translocation (Figure 7d), membrane blebbing, cell shrinkage (Figure 7e), significant increases in the average integrated nuclear fluorescence intensity (Figure 7f), and nuclear fragmentation (Figure 9d) observed in this study.

Several studies have reported the induction of cytoplasmic vacuolation, and markers associated with the atypical cell death mechanism, paraptosis [60,61,62]. It was therefore not unexpected to observe cytoplasmic vacuolation in this study (Figure 8a); however, it was the first report of cytoplasmic vacuolation induced in a breast cancer cell line using a cannabinoid combination. Paraptosis is characterized by cellular swelling and cytoplasmic vacuolation, resulting from the dilation of the endoplasmic reticulum and mitochondria, with the membrane of these cytoplasmic vacuoles often derived from the endoplasmic reticulum (Figure 9c) [63].

The exact molecular mechanism for the induction of paraptosis is not well established, although the most frequently reported induction signal is via the insulin-like growth factor receptor 1 (IGFR-1) [63,64]. IGFR-1 is overexpressed in the MCF-7 cells [15], providing a possible explanation for the presence of cytoplasmic vacuolation in the MCF-7 cell line only. Alternatively, a study by Hoa et al. [65] found that the activation of big conductance calcium-activated potassium (BK_Ca_) channels leads to the onset of ionic imbalance and cellular swelling associated with paraptosis. The forced activation of the BK channels results in potassium efflux, followed by an influx of sodium cations and water to restore ionic balance. This influx of water results in cellular swelling. Since the BK channels are located on the endoplasmic reticulum and mitochondria [65,66,67], this explains why these two organelles are specifically targeted during the induction of paraptosis, as seen in this study (Figure 9c,d). The excess intracellular Na^+^ is removed via ATP-dependent Na ^+^/H^+^ antiporter [68]. However, the physical disruption of the mitochondria impairs its ability to produce ATP, leading to an accumulation of intracellular Na^+^ and eventually the osmotic lysis of the cell [68]. The disruption of energy homeostasis is also associated with the induction of lipid droplet accumulation [69,70], linking the lipid accumulation induced by C6 (Figure 8b) to the initiation of paraptosis. Studies have also found that endoplasmic reticulum stress and the unfolded protein response (UPR) precede vacuole formation [60,71]. This supports the findings of this study, with significant increases in the GRP78 protein levels in the MCF-7 cells treated with C6 (Figure 9b). The induction of paraptosis by cannabinoids shows promise as an alternative treatment for apoptosis-resistant carcinomas, as well as the emerging field of targeting ion channels to treat cancer cells.

Conventional chemotherapeutic agents such as doxorubicin [72,73], paclitaxel [74,75], and tamoxifen [76,77] often induce adverse effects, which limit the dose that can be administered safely. Several studies have reported the limited induction of cytotoxicity by cannabinoids to the host in both in vitro and in vivo models [17,78,79,80], supporting the observations made in this study with regards to the MCF-10A cell line (Figure 6). It should be noted that conventional chemotherapeutic agents have been reported to induce cytotoxicity in the MCF-10A cell line at concentrations ranging from 1 to 15 µM [81,82], while the cannabinoid combination C6 induced no cytotoxicity in the MCF-10A cell line even at 40 µM. In this study, camptothecin treatment significantly reduced the MCF-10A cell viability, which supports the cytotoxic effects of chemotherapeutic agents on non-cancerous, viable cells. The anti-proliferative actions of cannabinoids are therefore not restricted by dose-limiting effects.

Cannabinoid signaling regulates several pathways in cancer and immune cells [83]. Cannabinoids reduce tumor proliferation by inhibiting the activation of the AKT [15,84], EGFR, ERK, and nuclear factor kappa beta (NF-κβ) signaling pathways [84,85]. NF-κβ is one of the key regulators of inflammation and tumor metastasis, survival, invasion, angiogenesis, and chemoresistance [86]. Cannabinoids also curtail tumor invasion and metastasis by inhibiting the secretion of matrix metalloproteinase (MMP)-2 [84,87] and MMP-9 [84,88], while increasing the tissue inhibitors of MMP-1 (TIMP-1) [89]. THC induces autophagy-mediated cell death as a result of stimulating the p8-regulated pathway. The stimulation of the p8 induces ceramide accumulation via the inhibitory effect of the tribbles homologue 3 (TRIB3) with the AKT/mTORC1 complex [51,52,90]. Therefore, cannabinoids exert their anti-tumor effects by modulating both the proliferative and inflammatory pathways. Like cannabinoids, other natural compounds such as curcumin, resveratrol, and calebin A modulate multiple signaling pathways associated with tumor cell proliferation, invasion, and metastasis. In addition, these compounds were evaluated in cultures mimicking the environment found in vivo and have shown promise in counteracting the synergistic crosstalk that occurs between cancer cells and the tumor microenvironment (TME) [91,92,93,94]. The modulation of the TME is important, since the TME plays a vital role in tumor development, invasion, and metastasis [83].

Conventional in vitro monolayer cell cultures inadequately represent the tumor environment found in vivo [95,96], highlighting a limitation of this study, where the effect of C6 on MCF-7 cells was not evaluated within a tumor microenvironment (TME). Although cannabinoids have shown a reduction in tumor proliferation within the TME [14,84,97], the evaluation of C6 within a TME remains important, since conflicting reports have been found. Cannabinoids can exert an immunosuppressive action against the anti-tumor immune cells found in the TME. Cannabinoid-induced immunosuppression incites tumor growth. This is mainly executed by reducing T cell proliferation and shifting the T helper profiles from a pro-inflammatory Th1 profile to an anti-inflammatory Th2 profile [98,99]. More specifically, this is accomplished through the decreased production of Th1 cytokines, such as interferon-γ (IFN-γ), interleukin-12, and interleukin-2; the reduced expression of the IFN-γ and IL-12 receptors; as well as an increased production of Th2-promoting cytokines (IL-10 and transforming growth factor-β) [100,101].

## 4. Materials and Methods

### 4.1. Reagents

All the cell lines were purchased from the American Type Culture Collection (Manassas, VA, USA). Dimethylsulfoxide (DMSO) was purchased from Merck (Darmstadt, Germany). Bradford reagent, sodium dodecyl sulphate (SDS) and the alkaline phosphatase conjugate substrate kit were purchased from Bio-Rad (Hercules, CA, USA). Annexin V-FITC and propidium iodide were purchased from BD Pharmingen (San Diego, CA, USA). Camptothecin, curcumin, hydrocortisone, bis-Benzamide H 33,342 trihydrochloride (Hoechst 33342), recombinant insulin, Leibovitz’s-L15, and trypsin were purchased from Sigma-Aldrich (St Louis, MO, USA). Cannabigerol, cannabinol, and cannabidiol were purchased from LGC (Teddington, UK). Δ^9^-tetrahydrocannabinol was purchased from Leco (St. Joseph, Michigan), Triton X-100 and mercaptoethanol were purchased from Fluka (North Carolina, NC, USA). Dulbecco’s Modified Eagle’s Medium (DMEM) high glucose with HEPES, DMEM/F12, foetal bovine serum, horse serum, and Roswell Park Memorial Institute (RPMI) 1640 were purchased from Biowest (Nuaillé, France). The mitochondrial staining kit (Cytopainter Orange) was purchased from Abcam (Cambridge, UK). ER Tracker^TM^ Green, Lysotracker^®^ Green DND-26, and lipidTOX^TM^ Green were purchased from Molecular Probes^®^, Life Technologies, and Thermo Fisher Scientific (Logan, UT, USA). 3-[4–dimethylthiazole-2-yl]-2,5 diphenyltetrazolium bromide (MTT) was purchased from Melford (Suffolk, UK). Epidermal growth factor was purchased from Life Technologies (Carlsbad, CA, USA). GRP78 (H-129) sc-13968 Rabbit polyclonal IgG was purchased from Santa Cruz (Dallas, TX, USA). *p*-p44/42 MAPK (T202/Y204) (D13.14.4E) XP rabbit mAb, goat anti-rabbit IgG conjugated to alkaline phosphatase, and goat anti-mouse IgG (Fc specific) alkaline phosphatase conjugate were purchased from Cell Signaling Technology (Danvers, MA, USA).

### 4.2. Cell Culture Conditions

The human breast cancer cell lines MCF-7 and MDA-MB-231 were cultured in DMEM high glucose and Leibovitz’s-L15, respectively, supplemented with 10% (*v*/*v*) FBS [102,103]. The non-cancerous human breast cell line, MCF-10A, was cultured in DMEM/F12, supplemented with 5% (*v*/*v*) horse serum, 20 ng/mL of epidermal growth factor, 10 µg/mL of insulin, and 0.5 µg/mL of hydrocortisone. The cultures were maintained at 37 °C in a humidified incubator with 5% CO_2,_ except for the MDA-MB-231 cells, which were incubated in an airtight container to limit the CO_2_ exchange.

### 4.3. Proliferation Assay

To evaluate the effect of individual cannabinoids (cannabidiol, cannabigerol, cannabinol, and Δ^9^-tetrahydrocannabinol) on the proliferation of breast cancer cell lines, MCF-7 and MDA-MB-231 were seeded in a 96-well plate at 1 × 10^4^ cells (100 µL/well) and left to attach overnight. The media was aspirated and replaced with 100 µL of the treatment at a concentration range of 16–64 µM. The working solutions were serially diluted to construct dose–response curves. After 48 h of treatment exposure, the cell viability was determined by replacing the spent media with 200 µL of MTT solution (0.5 mg/mL), followed by a 2 h incubation at 37 °C. The MTT solution was replaced with 200 µl of dimethylsulfoxide and the absorbance was recorded at 550 nm [104]. The GraphPad Prism^®^ curve fitting software was used to determine the IC_50_ value of the respective cannabinoids.

### 4.4. Combination Studies

#### 4.4.1. Constant Ratio Design

Cells (1 × 10^4^/well) were seeded into 96-well plates and left to attach overnight. Cannabinoids were combined at equipotent ratios of 1:1—i.e., the respective IC_50_:IC_50_ concentrations—thereby creating the following combinations: THC+CBG, THC+CBN, THC+CBD, CBG+CBN, CBG+CBD, and CBN+CBD. The cell viability was determined after 48 h of treatment exposure using the MTT assay. The nature of the drug:drug interactions were assessed by calculating the combination index using the median effect equation [26].

#### 4.4.2. Non-Constant Ratio Design

**Figure 10:**
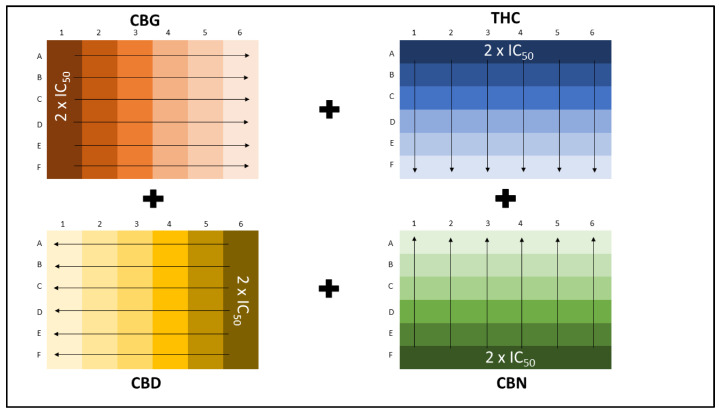
Illustration of the combination of four cannabinoids in a 96-well cell culture plate for the treatment of the breast cancer cell lines, MDA-MB-231 and MCF-7. Darkest color indicates the highest concentration, which was subsequently diluted in the direction illustrated.

Four cannabinoids were combined using the checkerboard assay, with each cannabinoid at six different concentrations. The checkerboard assay was designed with the maximum concentration of individual cannabinoids, being double their respective IC_50_ values, and combined to extend over a wide range of ratios (Figure 10).

#### 4.4.3. Determination of Combination Index

The Chou–Talalay method is based on the median effect equation derived from the mass action law principle. This general equation (equation 1) encompasses several equations, including the Michaelis–Menten, Hill, Scatchard, and Henderson–Hasselbalch equations. The equation uses a combination index as a measure of quantifying the drug–drug interactions, where CI = 1 represents an additive effect, CI < 1 signifies a synergistic effect, and CI > 1 signifies an antagonistic effect. The combination index can be calculated as follows:

CI = *[*_1_*/(Dx)*_1_ + _2_*/(Dx)*_2_] (1)

where _1_ and _2_ refer to the dose of each drug in the combination that is required to inhibit the cell growth by a given level, while (Dx)_1_ and (Dx)_2_ refer to the dose of the individual drugs required to induce the same level of cell growth inhibition and are determined by Equation 2:

*Dx = D_m_[fa/*(1 *– fa)]*^1/*m*^ (2)

where *Dm* refers to the potency (inhibitory concentration), *fa* refers to the fraction of the cell population affected by the drug, and *m* refers to the shape of the dose–effect curves.

### 4.5. Screening against Non-Cancerous MCF-10A Cell Line

Cells were seeded at a cell density of 1.5 × 10^4^ and incubated overnight for cell attachment. The cells were treated with the IC_90_ of C6 (40 µM; total concentration of all the cannabinoids comprising the C6 combination). Camptothecin (5.74 µM) was used as a positive control to induce growth inhibition. After treatment, the MTT assay was completed.

### 4.6. Mechanism of Cell Death

#### 4.6.1. Cell Seeding and Treatment

MCF-7 cells were seeded at a cell density of 1 × 10^4^ cells per well (100 µL) and left overnight to attach. The cells were treated with the IC_90_ concentration of C6 for 48 h at 37 °C.

#### 4.6.2. Imaging and Analysis

Images of cells stained with fluorescent dyes were acquired using the ImageXpress Micro XLS Widefield High-Content Analysis system (Molecular Devices^®^, San Jose, CA, USA) and analyzed with the MetaXpress^®^ High-Content Image Acquisition and Analysis Software version 6.01 (Molecular Devices^®^, San Jose, CA, USA). Assays were conducted in 96-well plates and 9 sites per well were imaged, covering an area of approximately 63.5% of the total well area. The number of cells imaged was dependent on the cytotoxicity of the treatment [105].

##### Cell Cycle Analysis

After treatment, the spent media was aspirated and the cells were stained with a 1 × binding buffer stock (0.1M HEPES, pH 7.4; 1.4M NaCl; 25mM CaCl2), supplemented with 5% (*v*/*v*) Annexin V-FITC (BD PharmingenTM) and Hoechst 33,342 at a final concentration of 1 µg/mL in the dark at 37 °C for 15 min. Images were acquired using the 4′,6′-diamidino-2-phenylindole (DAPI) and fluorescein isothiocyanate (FITC) filters.

##### Phosphatidylserine Translocation

The cells were stained according to the manufacturer’s instructions. Prior to image acquisition, propidium iodide was added (final concentration of 1 µg/mL) to the Annexin V/Hoechst stain. Images were acquired using the DAPI, FITC, and Texas Red filter sets.

##### Induction of Autophagy-Related Processes

The cells were stained according to the manufacturer’s instructions, with slight modifications. The cells were co-stained with Lysotracker^®^ Green (75 nM) and 1 µg/mL of Hoechst 33,342 for 30 min at 37 °C, followed by image acquisition using the DAPI and FITC filters.

##### Lipid Accumulation

After treatment, the spent media were removed, and the cells were stained with HCS LipidTOXTM Green (1:200) and 1 µg/mL of Hoechst 33,342 and incubated in the dark for 30 min at 37 °C. Images were acquired using the DAPI and FITC filters.

##### Endoplasmic Reticulum Staining

After treatment, the spent media were aspirated and the cells were stained with ER-TrackerTM (1 µM) and 1 µg/mL of Hoechst 33,342 for 15 min at 37 °C, which was subsequently replaced with fresh media and imaged using the DAPI and TRITC filters.

#### 4.6.3. Mitochondrial Dilation

The cells were seeded at a 1 × 10^3^ cells per well in a µ-slide (Ibidi, Martinsried, Germany). After overnight attachment, the cells were treated with C6 (40 µM). The spent media were removed, and cells were stained using the CytoPainter Orange mitochondrial staining kit (1:500), as per the manufacturer’s instructions. After a 30 min incubation, the stain was replaced with binding buffer and the cells were imaged with the Zeiss LSM510 Meta laser scanning confocal microscope using the DAPI and TRITC filter sets.

#### 4.6.4. Western Blotting Analysis

After cell treatment, the media were aspirated and the cells were washed with ice-cold phosphate buffered saline, followed by a 10-min incubation in lysis buffer (50 mM of Tris-HCl, pH 7.4; 2 mM of EDTA, 1% (*v*/*v*) Triton X-100). The cells were lifted using a cell scraper and the lysates were vortexed for 10 s and aliquoted for storage at −20 °C. When required, the protein concentrations of the lysates were determined using the Bradford assay, and equal protein concentrations were electrophoresed on a 10% SDS-PAGE [106]. The protein was transferred to a PVDF membrane using electroblotting with the Biorad trans-blot transfer system at 20 V for 20 min. The PVDF membrane was blocked with 5% (*w*/*v*) milk powder and the respective primary antibodies were added (1:1000) and incubated overnight at 4 °C. This was followed by incubation with the anti-rabbit alkaline phosphatase conjugated secondary antibody (1:20,000) for 3 h at 4 °C. Bands were detected using the Bio-Rad alkaline phosphatase substrate kit [107]. The protein expression was normalized to the total protein. Replicate gels from independent experiments were stained with Coomassie Brilliant Blue and densitometric analysis was used to quantify the aggregate signal of each lane with the Image J 1.52a software (USA). This value was used as the total protein of the sample loaded per lane.

### 4.7. Statistical Analysis

All the experiments were performed in triplicate (*n* = 3). The values obtained were expressed as the mean ± standard error unless otherwise stated. An analysis of variance with post hoc Tukey test was conducted to analyze the significance between various treatments, relative to the vehicle controls. Statistics was calculated using GraphPad Prism^®^ 5 version 5.01 (GraphPad Software, San Diego, California, USA).

## 5. Conclusions

To date, the combination of four cannabinoids as a potential cancer treatment has not been reported. With limited evidence of cytoplasmic vacuolation and paraptosis induction by phytocannabinoids, this study paves the way for the in-depth mechanistic evaluation of cannabinoid combinations and their alternative anti-proliferative mechanisms. Cannabinoids can effectively target different subtypes of breast cancer and display several properties that aid in minimizing the early onset of drug resistance, such as dose reduction, improved uptake across the cell membrane, and curtailing drug efflux through ATP-binding cassette transporters. Furthermore, the high selectivity cannabinoids display towards cancer cells and their alleviation of the side-effects associated with cancer and cancer treatment increase the promise for cannabinoid use in standardized cancer treatment. This study also demonstrated the possibility of crosstalk between the three cell death pathways with markers commonly associated with each cell death pathway, in addition to markers associated with all three pathways. It is yet to be confirmed if these pathways are activated independently or modulated via a common signaling intermediate.

## 6. Patents

Please note that a patent has been filed from the work reported in this manuscript.

## Figures and Tables

**Table 1:** Inhibitory concentrations of Δ^9^-tetrahydrocannabinol, cannabigerol (CBG), cannabinol (CBN), and cannabidiol (CBD) at the selected effect levels.

Inhibitory Concentration (µM)	MDA-MB-231			MCF-7		
	IC_50_	IC_75_	IC_90_	IC_50_	IC_75_	IC_90_
THC	30.13 ± 3.94	30.57 ± 1.05	35.72 ± 2.32	40.14 ± 4.68	48.37 ± 3.86	58.55 ± 3.28
CBG	28.40 ± 4.63	33.40 ± 6.34	39.34 ± 8.54	31.45 ± 2.28	37.85 ± 2.61	45.60 ± 3.30
CBN	23.22 ± 2.62	28.22 ± 2.12	34.84 ± 4.40	28.19 ± 2.98	32.21 ± 3.92	36.84 ± 5.14
CBD	13.82 ± 1.96	25.80 ± 1.40	49.20 ± 4.48	20.62 ± 1.66	28.12 ± 3.12	38.54 ± 5.68

**Table 2:** Inhibitory concentrations of combinations consisting of two cannabinoids.

Inhibitory Concentration (µM) of Combination	MDA-MB-231			MCF-7		
	IC_50_	IC_75_	IC_90_	IC_50_	IC_75_	IC_90_
THC and CBN	73.85 ± 9.52	89.04 ± 12.21	80.25 ± 14.65	28.16 ± 1.95	33.99 ± 1.70	34.06 ± 8.71
THC and CBD	52.12 ± 4.02	65.97 ± 2.91	71.29 ± 13.90	20.23 ± 1.17	35.09 ± 6.23	53.27 ± 14.79
CBG and CBN	40.29 ± 5.54	48.32 ± 6.65	65.41 ± 7.40	32.91 ± 6.68	50.56 ± 5.30	79.33 ± 1.05
CBG and CBD	31.88 ± 4.21	38.09 ± 5.26	45.59 ± 6.94	31.02 ± 4.16	48.59 ± 4.00	72.88 ± 3.88
CBN and CBD	31.62 ± 4.77	44.94 ± 9.61	64.35 ± 17.80	31.22 ± 6.26	41.48 ± 5.27	55.44 ± 4.27
